# Graphene-enhanced NiMn_2_O_4_ composite for high-performance supercapacitor applications

**DOI:** 10.1039/d6ra02865a

**Published:** 2026-07-10

**Authors:** Pakeeza Aymen Nawaz, Muhammad Boota, Ali Mujtaba, Abdullah Almohammedi, Fatimah M. Alzahrani, Sadia Nazir, Ghalib ul Islam, M. I. Khan, Munawar Iqbal, Arif Nazir

**Affiliations:** a Department of Physics, The University of Lahore Lahore 54000 Pakistan; b International Center for Interdisciplinary Research in Sciences (ICIRS), The University of Lahore Lahore 54000 Pakistan muhammad.boota@phys.uol.edu.pk; c Department of Physics, Faculty of Science, Islamic University of Madinah Madinah 42351 Saudi Arabia; d Department of Chemistry, College of Science, Princess Nourah bint Abdulrahman University P.O. Box 84427 Riyadh 11671 Saudi Arabia; e Department of Physics, Division of Science and Technology, University of Education Lahore 54770 Pakistan; f Zero Emission Technologies Innovation Center, University of Tabuk Tabuk 47913 Saudi Arabia; g Department of Chemistry, Faculty of Science, University of Tabuk Tabuk 47913 Saudi Arabia; h Department of Chemistry, The University of Lahore Lahore 54000 Pakistan

## Abstract

The growing global demand for efficient and sustainable energy storage solutions has highlighted the need for advanced materials to address the energy crisis. In this work, the synthesis and electrochemical performance of a NiMn_2_O_4_ and Graphene@NiMn_2_O_4_ (1 : 1) composite for supercapacitor applications has been studied. The novelty lies in the integration of graphene with NiMn_2_O_4_, enhancing its electrochemical properties. X-ray diffraction (XRD) analysis revealed that NiMn_2_O_4_ exhibited a crystallite size of 21.4 nm, while the Graphene@NiMn_2_O_4_ composite showed an increased crystallite size of 28.1 nm. Raman spectroscopy confirmed the successful hybridization of graphene with NiMn_2_O_4_, exhibiting D and G bands that indicate increased defect density beneficial for charge storage. Scanning electron microscopy (SEM) analysis demonstrated the hierarchical morphology of Graphene@NiMn_2_O_4_, with well-dispersed NiMn_2_O_4_ nanoparticles on graphene sheets, which promoted better porosity and electrolyte penetration. Electrochemical results from cyclic voltammetry (CV) showed that Graphene@NiMn_2_O_4_ exhibited superior capacitance, with a highest capacitance of 301 F g^−1^ at 0.8 A g^−1^, compared to 229 F g^−1^ for pure NiMn_2_O_4_. Galvanostatic charge–discharge (GCD) tests showed a considerable improvement in energy density for Graphene@NiMn_2_O_4_, reaching 3.8 Wh kg^−1^ at 0.8 A g^−1^, compared to 2.9 Wh kg^−1^ for NiMn_2_O_4_. Electrochemical impedance spectroscopy (EIS) exhibited reduced charge transfer resistance (*R*_ct_) and enhanced ion diffusion coefficient for the composite indicating superior charge transport. These findings highlight the potential of Graphene@NiMn_2_O_4_ as a promising material for high-performance energy storage devices. Future work will focus on optimizing the composite structure for commercial supercapacitor applications.

## Abstract 2

Graphene modified NiMn_2_O_4_ showed improved crystal size, with reduced charge transfer resistance, increased ion diffusion coefficient and improved capacitance.

## Graphical abstract

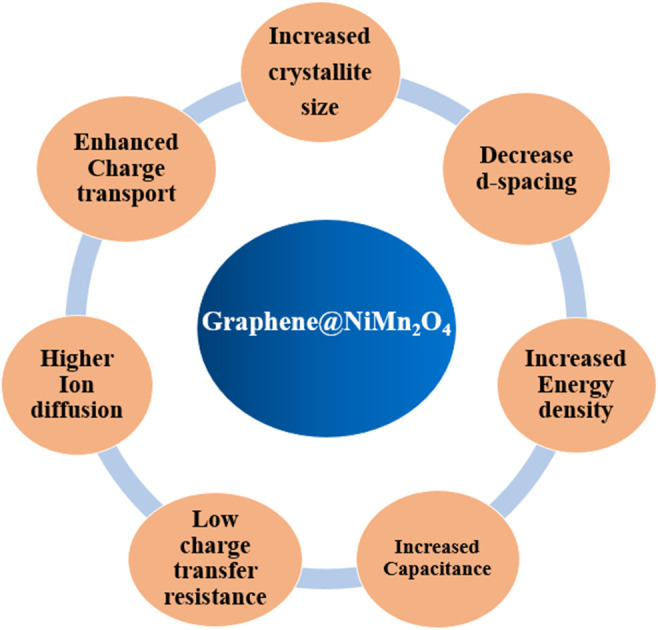

## 1. Introduction

The increasing worldwide demand for energy and the depletion of fossil resources have increased the urgency of substitute sustainable energy storage technologies.^1,2^ Renewables like solar and wind energy are also hindered by their intermittency and need to be stored effectively and resiliently to be widely adopted.^3^ Because of their scalability, adaptability and compatibility with RE sources, electrochemical systems, especially supercapacitors, have received interest.^4^ Supercapacitors can be categorized as electric double-layer capacitors (EDLCs) and pseudocapacitors. Their advantages include rapid charge/discharge performance, higher power density and long-life duration.^5^ The charge storage in EDLC is by electrostatic adsorption, providing high power density and stability, but limited energy density.^6^ Despite the ability to achieve increased energy density through faradaic redox processes, the effectiveness of pseudocapacitors depends on the material design aspects such as conductivity, ion diffusion and structural integrity.^7^ Transition metal oxides (TMOs) are very desirable because of their outstanding theoretical capacitance, chemical stability and ecologically friendly properties.^8^ The main reason for the attractiveness of manganese-based oxides is that they are cheap, abundant in nature and environmentally safe.^9^ However, conventional manganese oxides often suffer from poor electrical conductivity and degradation upon cycling.^10^ Mixed-metal spinel oxides provide a possible answer to these problems, not only by providing a structurally stable three-dimensional framework, but also by providing superior electronic conductors.^11^ In this context, spinel nickel manganese oxide (NiMn_2_O_4_) has become an important material of interest because it offers a number of charge storage sites through the redox activity of the Ni^2+^/Ni^3+^ and Mn^3+^/Mn^4+^ couples and has a high degree of structural stability. Although these benefits, the low intrinsic conductivity together with the partial utilization of active sites remains a limitation for the electrochemical performance of pristine NiMn_2_O_4_.^12^

The kinetic and electrical limitations of pseudocapacitive materials have been overcome by the coupling of conductive carbon frameworks and transition metal oxides.^13^ The extraordinary electrical conductivity, extremely high specific surface area, mechanical flexibility, and chemical durability distinguishes graphene from other carbonaceous materials.^14^ Graphene has these properties, which make it possible to use it as an additive for conductive materials and as an active medium for charge accumulation and rapid electron transport.^15^ As a conductive scaffold, graphene can also form heterostructure surfaces with metal oxides to enable strong electronic coupling and a reduced interfacial resistance.^8,16^ In the graphene–spinel oxide heterostructures, graphene is used for the electrical double layer capacitance, and rapid electron transport, while the metal oxide is used for faradaic pseudo-capacitance.^17^ Heterointerface engineering for NiMn_2_O_4_ is especially beneficial because it promotes charge transfer kinetics, reduces agglomeration of particles, and promotes the spinel lattice's stability over long cycles, resulting in higher rate capability and cycling stability.^12^

Recent studies have demonstrated that Ni–Mn–based oxides as well as graphene hybrids can be effective for high-performance supercapacitors. Raj *et al.* were the first to report spinel NiMn_2_O_4_ nanosheets on a nickel foam (3-D). The synthesized NiMn2O4 NSs@NF depicts a specific capacitance of 770.7 F g^−1^, a rate capability of 84.6%, and incredible capacitance retention of 94.5% after 10 000 cycles.^18^ Wei *et al.* fabricated NiCo_2_O_4_/rGO/NF by an aqueous co-precipitation-hydrothermal method. It was used to fabricate a binder-free integrated electrode for supercapacitors, giving an exceptional specific capacitance of 2863.4 F g^−1^ (1503.3 C g^−1^) at 1 A g^−1^, an outstanding rate performance of 2335.2 F g^−1^ at 20 A g^−1^ and having a stability retention of 91.7% after 5000 cycles.^19^ Polat *et al.* demonstrated the synthesis of the graphene-doped MnO_2_-coated carbon cloth electrodes. The electrochemical investigation revealed diffusion-controlled charge storage mechanism, achieving a specific capacitance of 513 F g^−1^ (205 F cm^−3^–1027 mF cm^−2^) at the current density of 1 A g^−1^.^20^ Muthu *et al.* studied the rGO/NiMoO_4_ nanocomposite prepared by a hydrothermal method and then annealed at 450 °C. The capacitance of the rGO/NiMoO_4_ composite was 680 F g^−1^ and showed 68% capacitance retention after being cycled 4000 times at 3 A g^−1^.^21^

A NiMn_2_O_4_/rGO nanocomposite was synthesized employing a simple sol–gel method and was shown to be an efficient catalyst by Sandhiya *et al.* Used in making a supercapacitor. This showed a specific capacitance of 710 F g^−1^ as compared to NMO (254 F g^−1^).^22^ Furthermore, the SSC device showed a 90% capacitance retention after 10 000 cycles at a coulombic efficiency of 99% when tested at 5 A g^−1^, making it capable of being used for high-energy supercapacitors. Li *et al.* have successfully fabricated NiMn_2_O_4_ NSs@rGO by a simple thermal treatment and co-precipitation method. The synthesized NiMn_2_O_4_ NSs@rGO nanocomposite exhibits excellent energy storage performance with a specific capacitance of 1243 F g^−1^ at a current density of 3 A g^−1^ and maintains 80.8% capacitance after 5000 cycles after assessment.^23^ A NiMoO_4_/g-C_3_N_4_ composite was prepared by the hydrothermal method by Thiagarajan *et al.* The results show that this NiMoO_4_/g-C_3_N_4_ nanocomposite has a better MSC value of 510 F g^−1^ than the pristine NiMoO_4_ with a value of 203 F g^−1^.^24^

The integration of graphene with NiMn_2_O_4_ presents a novel approach to enhancing the electrochemical performance of supercapacitor materials. The unique combination of graphene's high conductivity and the redox activity of NiMn_2_O_4_ creates a synergistic effect, improving ion diffusion and also charge transfer. The addition of graphene increased the crystallite size of NiMn_2_O_4_, facilitating better ion adsorption and diffusion, while also improving the overall conductivity of the composite. This results in improved electrochemical stability and enhanced capacitance. Graphene@NiMn_2_O_4_ exhibited a remarkable increase in capacitance, achieving 301 F g^−1^ at 0.8 A g^−1^ compared to 229 F g^−1^ for pure NiMn_2_O_4_, demonstrating the composite's superior performance. Furthermore, the *R*_ct_ of Graphene@NiMn_2_O_4_ was significantly lower (0.22 Ω) than that of NiMn_2_O_4_ (2.87 Ω), indicating a more efficient electron transfer process. These improvements are attributed to the enhanced surface area, conductivity, and structural integration provided by graphene. Future work will focus on optimizing the composite and exploring its scalability for practical energy storage applications.

## 2. Experimental details

### 2.1 Materials required

Several materials were needed to synthesize NiMn_2_O_4_ and Graphene@NiMn_2_O_4_. The nickel and manganese to prepare the solutions were in the form of nickel nitrate hexahydrate, Ni(NO_3_)_2_·6H_2_O (99%) and manganese nitrate tetrahydrate, Mn(NO_3_)_2_·4H_2_O (99%). Graphene was used as a starting material for the synthesis of Graphene@NiMn_2_O_4_. Fresh leaves of Neem were used for the preparation of an aqueous extract, used as a reducing and stabilizing agent.^25^ Neem extract was prepared by boiling 60 g of neem leaves in 200 mL of deionized water for 30 minutes.^26^ Deionized water and ethanol (C_2_H_5_OH 99%) were used for washing and purification processes, and hydrochloric acid (HCl 37%) and sodium hydroxide (NaOH 99%) were used for the pH adjustment of the solutions when necessary. All the chemicals used were reagent grade and used as received.

### 2.2 Synthesis of materials

**Fig. 1:**
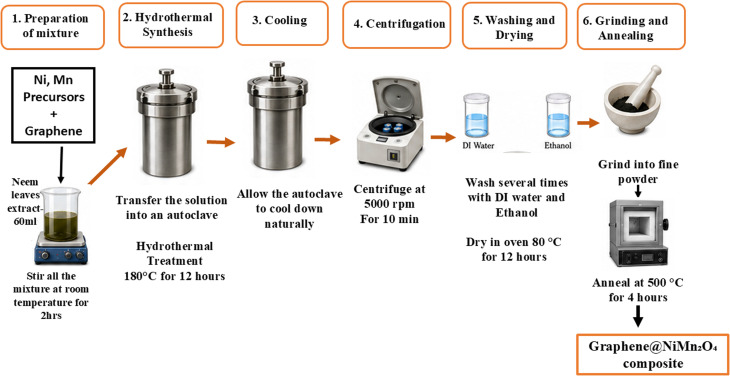
Step-wise hydrothermal synthesis of Graphene@NiMn_2_O_4_.

A solution of 1 M Ni(NO_3_)_2_·6H_2_O (17.448 g in 60 mL neem leaves exract), with a 2 M Mn(NO_3_)_2_·4H_2_O (30.121 g) were prepared. A neem leaf extract was prepared by boiling 40 g of neem leaves in 200 mL of deionized water for 1 hour, then filtering to obtain a clear extract.^27^ The prepared Ni and Mn solutions were mixed to prepare NiMn_2_O_4_. The solution of Ni(NO_3_)_2_·6H_2_O and Mn(NO_3_)_2_·4H_2_O was prepared by adding 60 mL of neem extract.^28^ The mixture was stirred at room temperature for 2 hours to ensure the uniformity of integration of the ingredients.^29^ This solution was then transferred into an autoclave, and the hydrothermal synthesis was carried out at 180 °C at autogenous pressure for 12 hours.^30^ After the reaction, the autoclave was allowed to cool to ambient temperature. This was then centrifuged at 5000 RPM for 10 minutes to isolate the product. Precipitate was collected, carefully washed with deionized water and ethanol to remove any trace of contaminants, and dried in an oven set at 80 °C for 12 hours. It was dried and then ground into a powder using a mortar and pestle. The material was also further fired in a furnace at 500 °C for 4 hours to get the pure NiMn_2_O_4_ powder.^31^ The precursor solutions were formulated and the synthesis of NiMn_2_O_4_ began. The Ni(NO_3_)_2_·6H_2_O (1 M), Mn(NO_3_)_2_·4H_2_O (2 M), and 2.149 g of graphene (G) were added into 60 mL of the prepared neem leaf extract. The mixture was stirred continuously for 2 h at room temperature to achieve uniform dispersion of the graphene oxide and to get a uniform mixture of all the precursors.^32^ The prepared solution was then placed in a Teflon-lined stainless-steel autoclave and the pressure was raised up to 180 °C for 12 hours of hydrothermal treatment. The product obtained was collected after natural cooling at room temperature by centrifugation at 5000 rpm for 10 minutes.^33^ The precipitate was thoroughly washed several times with deionized water and ethanol in order to eliminate the remaining impurities and unreacted species. Finally, the material obtained was dried in an oven at 80 °C for 12 hours, ground to a fine powder, and then annealed at 500 °C for 4 hours to get the final Graphene@NiMn_2_O_4_ composite (Fig. 1).^34^

### 2.3 Electrode fabrication

For the electrochemical tests, the electrodes were prepared by mixing a 10% (mass) amount of Super P carbon black (conductive agent) and 10% (mass) polyvinylidene fluoride (PVDF) (binder) with 80% (mass) of NiMn_2_O_4_ or Graphene@NiMn_2_O_4_. The active ingredient and carbon black were mixed in powder form and the PVDF binder was solubilized in *N*-methyl-2-pyrrolidone (NMP) to form a slurry, which was then added to the powder mixture of the above two components.^35^ The resultant paste was meticulously blended and applied on a copper foil current collector with a doctor blade method. The coated electrodes were dried in the oven at 60 °C for 12 hours to remove any solvent residue from the coated surface.^36^ The mass of the active material deposited on the electrode was ∼5 mg. The dried Cu foil was cut into 12 mm diameter round discs for the fabrication of working electrodes. For electrochemical evaluations, a three electrode setup was used.^37^ The electrolyte was a 1 M KOH solution.

### 2.4 Characterizations

Several methods were used to investigate the structure and morphology of the materials synthesized. The NiMn_2_O_4_ and Graphene@NiMn_2_O_4_ materials were investigated using XRD on a Bruker D8 Advance X-ray diffractometer. The Raman spectra were recorded using the Raman spectrometer Horiba Scientific LabRAM HR Evolution with the use of 532 nm laser excitation. The surface morphology and surface microstructure of the materials were analyzed by SEM with use of FEI Quanta 250 FEG SEM. The characterization of CV, GCD, and EIS was done using an Electrochemical CS350 M workstation. CV was used to perform electrochemical characterizations with a potential range of 0–0.7 V *vs.* Ag/AgCl and a scan rate of 5–50 mV s^−1^. Current densities ranging from 0.8 to 2.0 A g^−1^ were used for GCD testing. The voltage range of these studies was fixed between 0–0.7 V (against Ag/AgCl). The electrodes have been evaluated by EIS in the frequency range of 0.1 Hz to 100 kHz to determine the charge transfer resistance and ion diffusion properties of the electrodes.

## 3. Results and discussion

### 3.1 XRD analysis

**Fig. 2:**
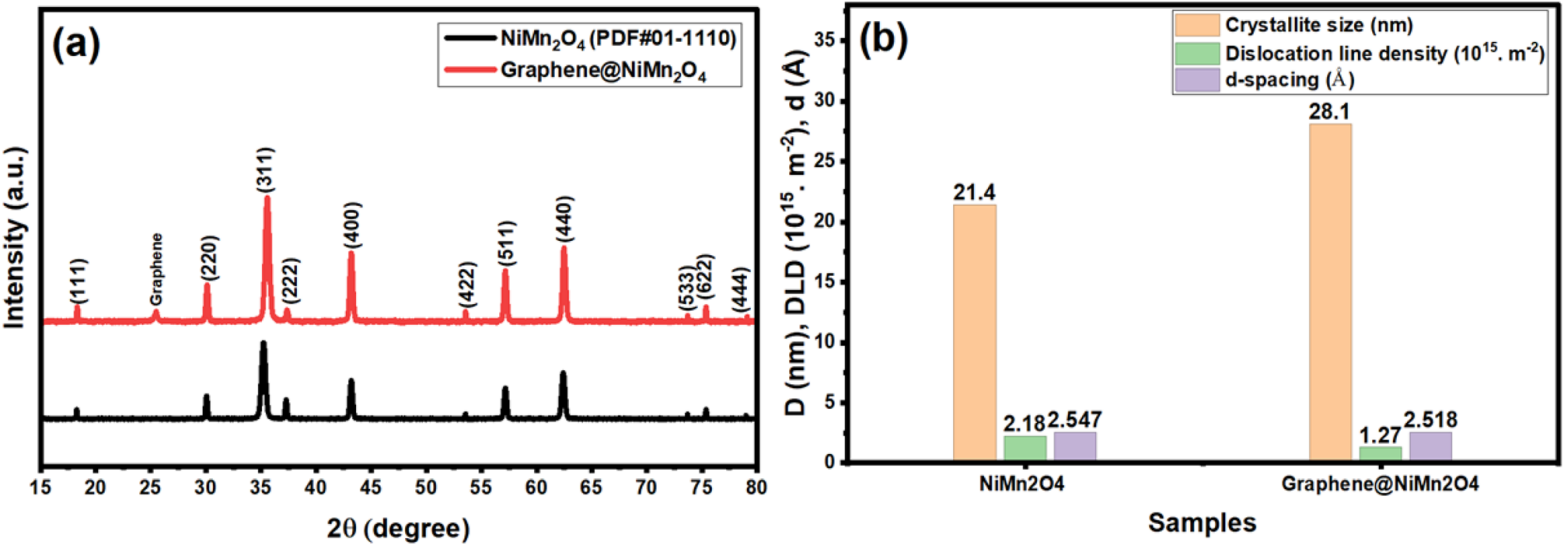
(a) depicts the XRD pattern and (b) crystallite size, *d*-spacing, and DLD of NiMn_2_O_4_ and NiMn_2_O_4_@Graphene.

Phase composition, crystal structure and lattice disorder, which have direct influence on electronic transport and ion diffusion, are detected by XRD.^38^ The XRD structures of NiMn_2_O_4_, and Graphene@NiMn_2_O_4_ composite are shown in Fig. 2(a). The NiMn_2_O_4_ sample shows well-defined and clear diffraction peaks at 2*θ* corresponding to (111), (220), (311), (222), (400), (422), (511), (440), (533), (622), and (444) planes, which confirms the formation of the NiMn_2_O_4_ crystalline cubic spinel phase.^41^ The sharpness of these peaks indicates a high degree of atomic ordering, leading to stable redox reactions with well-defined Ni^2+^/Ni^3+^ and Mn^3+^/Mn^4+^ redox couples.^42^ Even after modifying the crystal structure, all the characteristic peaks of NiMn_2_O_4_ are clearly observed in the Graphene@NiMn_2_O_4_ composite, in addition to a diffraction peak at 2*θ* ≈ 25.6°, related to the (220) plane of graphene, is due to stacked π–π stacked graphene layers.^39^ The wide characteristic suggests the reduced stacking order and reduced thickness of the crystallites, thereby increasing surface accessibility.^40^ All the diffraction planes measured are in good agreement along with the standard spinel NiMn_2_O_4_ phase, with PDF# 01-1110, which confirms the absence of secondary contaminants and phase purity.^43^ The formulas below are for the crystallite size (*D*), *d*-spacing (*d*), and dislocation line density (*δ*), which were employed to determine the structural parameters:^44–46^1
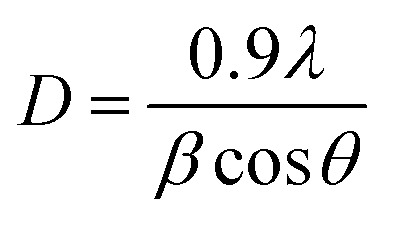
2
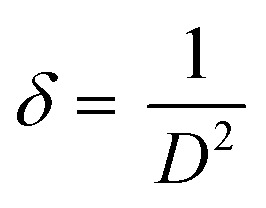
3
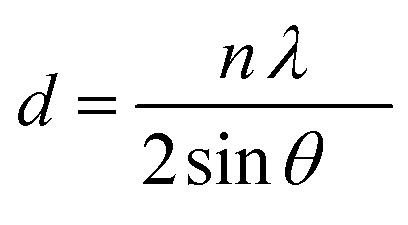


In this, *β* is the full width at half maximum (FWHM), *λ* is the wavelength of the X-ray, and *θ* is the Bragg angle. Fig. 2(b) presents the crystallite size (*D*), dislocation line density (DLD), and interplanar spacing (*d*-spacing) of pristine NiMn_2_O_4_ and Graphene@NiMn_2_O_4_ calculated from XRD data (Table 1). The incorporation of graphene increases the crystallite size from 21.4 nm for NiMn_2_O_4_ to 28.1 nm for Graphene@NiMn_2_O_4_, indicating improved crystal growth and enhanced structural ordering.^47,48^ Meanwhile, the dislocation line density decreases from 2.18 × 10^15^ m^−2^ to 1.27 × 10^15^ m^−2^, suggesting a reduction in lattice imperfections and improved crystallinity after graphene integration. The *d*-spacing exhibits a slight decrease from 2.547 Å to 2.518 Å, which may be attributed to interfacial interactions and lattice rearrangement induced by the graphene sheets.^49^ The reduced defect density and enhanced crystallinity facilitate more efficient electron transport and lower internal resistance during electrochemical processes. Furthermore, the conductive graphene framework provides interconnected pathways for rapid charge transfer while maintaining structural stability. These structural improvements are expected to accelerate ion diffusion kinetics, enhance charge storage efficiency, and contribute significantly to the superior electrochemical performance of the Graphene@NiMn_2_O_4_ electrode for advanced supercapacitor applications.^50^

**Table 1:** Structural parameters of graphene, NiMn_2_O_4_ and Graphene@NiMn_2_O_4_

Parameter	Graphene (theoretical)	NiMn_2_O_4_	Graphene@NiMn_2_O_4_
Crystalline size (nm)	40–60 [ref. 51]	21.4	28.1
*d*-Spacing (Å)	3.35 [ref. 52]	2.547	2.518
Dislocation line density (10^15^)	—	2.18	1.27
Lattice constant (Å)	2.46 [ref. 53]	8.35	8.39
Volume unit cell (Å)^3^	—	583.8	590.8

### 3.2. Raman analysis

**Fig. 3:**
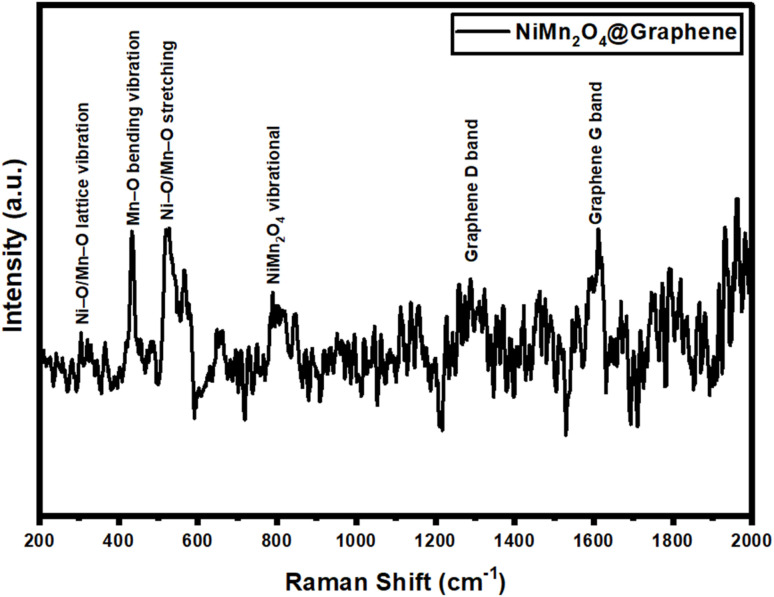
Raman spectra of Graphene@NiMn_2_O_4_.

The Raman spectra (Fig. 3) of NiMn_2_O_4_@Graphene show sharp peaks at about 247, 432, 521 and 786 cm^−1^, indicating the spinel NiMn_2_O_4_ structure, thereby confirming its phase development.^49,54^ The notable D band (∼1302 cm^−1^) and G band (∼1598 cm^−1^) confirm the existence of graphene and suggest structural flaws and graphitic ordering, respectively.^49^ The intensity ratio of the D and G band is proportional to the density of defects, which is beneficial for electrochemical activity. The peaks of metal oxide and graphene are present, which confirms the formation of the hybrid. In this synergistic system, the electron transport and charge storage efficiency is improved.^55^

### 3.3. Surface morphology

**Fig. 4:**
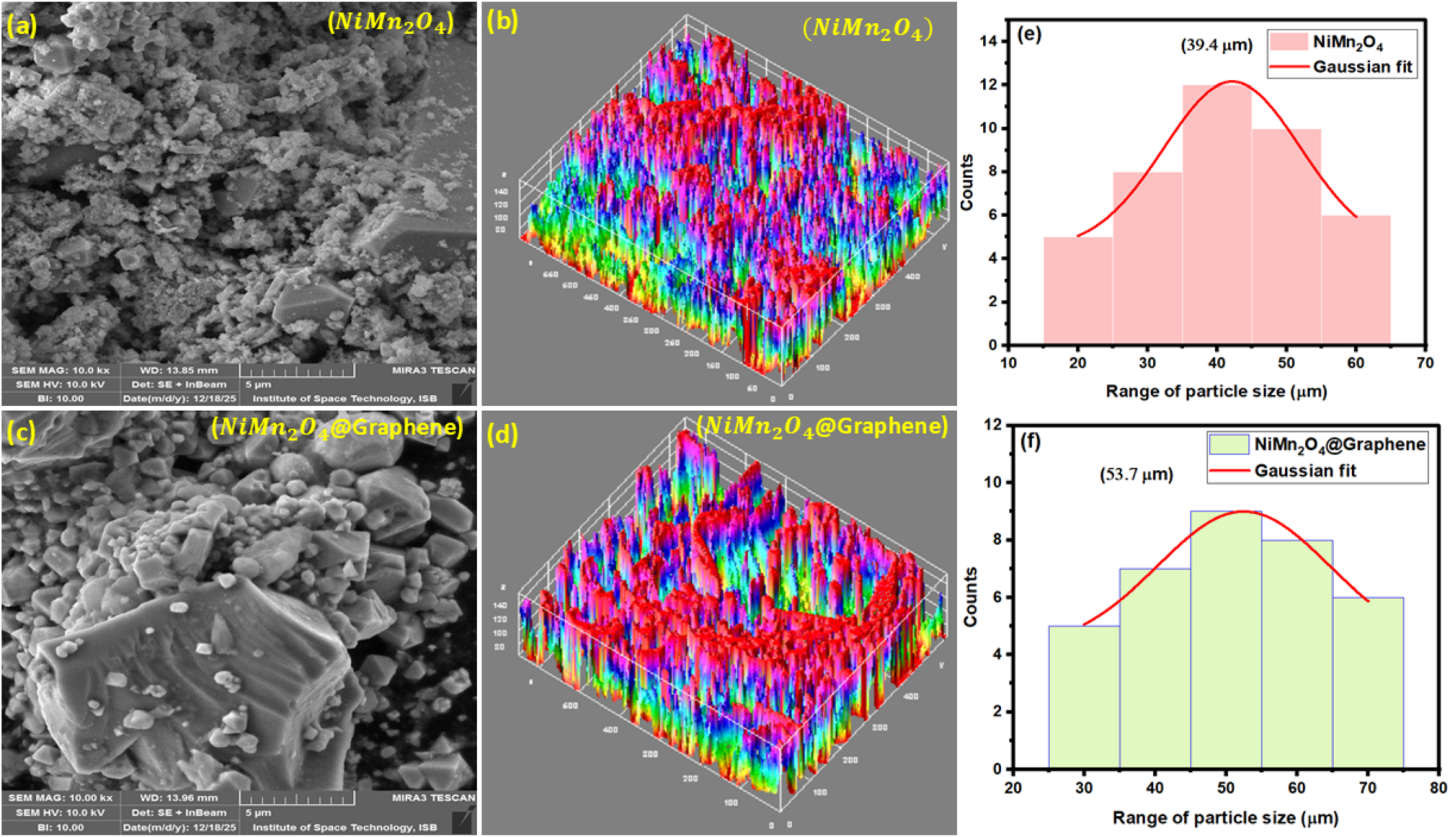
(a and c) 2D SEM images, (b and d) 3D SEM images (e and f) particle size distribution of NiMn_2_O_4_, and NiMn_2_O_4_@Graphene.

SEM images are shown in Fig. 4(a–f) to show the morphological transition from pure NiMn_2_O_4_ to NiMn_2_O_4_@Graphene. Pure NiMn_2_O_4_ has an extremely agglomerated structure with rough surfaces and unevenly shaped particles, which may hinder the effective utilization of the electrolyte (Fig. 4(a)).^43^ A 3D topographical view (Fig. 4(b)) shows reasonably dense features on the surface with little dispersion of the voids. The NiMn_2_O_4_@Graphene composite, on the other hand, has a more distinct and interconnected morphology (Fig. 4(c)).^56^ The NiMn_2_O_4_ particles show good anchoring on graphene sheets and thus form a hierarchical structure. Graphene helps to reduce agglomeration and improve dispersion of particles.^57^ The 3D SEM image shown in Fig. 4(d) clearly shows the increased surface roughness, numerous voids and higher topographical diversity compared to the pure NiMn_2_O_4_, confirming the creation of a porous structure.^58^ Such a porous structure is beneficial to energy storage because it allows good penetration by the electrolyte and rapid diffusion of ions.^59^ The particle size distribution for SEM images was measured using image software and column histograms were plotted and Gaussian fitting was applied to determine the average grain size. The histograms of particle size distribution (Fig. 4(e and f)) show that the average size of particles increased from 39.4 µm for NiMn_2_O_4_ to 53.7 µm for NiMn_2_O_4_@Graphene, which is proof of the formation of composite structures. The improved porosity, surface area and structural integration of NiMn_2_O_4_@Graphene are expected to significantly enhance electrochemical performance.^60^

### 3.4. Electrochemical analysis

**Fig. 5:**
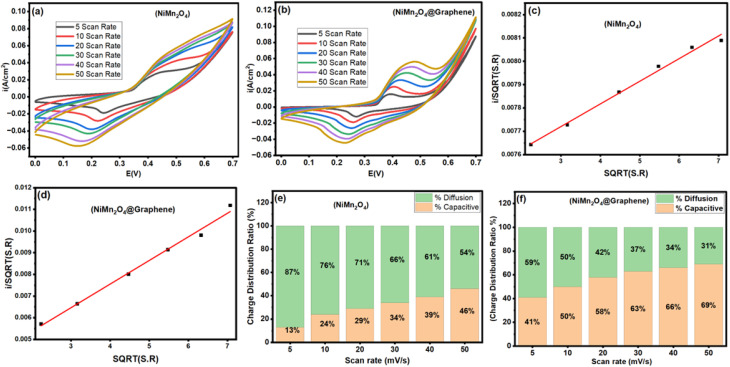
(a and b) CV cycles, (c and d) verses between SQRT (S.R) *vs.* i/SQRT(S.R) (e and f) capacitive and diffusion (%) calculated by Dunn method of NiMn_2_O_4_, and NiMn_2_O_4_@Graphene.

The electrochemical behaviors of Graphene@NiMn_2_O_4_ in KOH electrolyte were studied by cyclic voltammetry (CV) and the results are presented in Fig. 5. The reactions and mechanisms of this substance are of crucial importance in order to understand the electrochemical processes. The electrochemical reaction process of NiMn_2_O_4_ in KOH is as follows:^61,62^

In the reduction process:NiMn_2_O_4_ + 4e^−^ + 4OH^−^ → NiO + 2MnO_2_ + 2H_2_OIn the oxidation process:NiO + 2MnO_2_ + 2H_2_O → NiMn_2_O_4_ + 4e^−^ + 4OH^−^

Add graphene to enhance the total electrochemical activity of the composite material as well as the surface area and conductivity. The graphene part enhances the speed at which electrons move through the material during charging and discharging, which leads to better energy storage properties.^63^ The cyclic voltammetry (CV) peaks of NiMn_2_O_4_ at various scan rates are shown in Fig. 5(a). Redox reactions happen in the material are indicated by the peaks in the CV curves. With an increase in scan rate, the peak current rises, and indicating diffusion-controlled processes.^64^ The current response is strongly affected by the ion transport as shown by the redox peaks of NiMn_2_O_4_. With higher scan rates, this increase in the peak current suggests that diffusion is the rate-determining step at these rates.^65^ The CV peaks for Graphene@NiMn_2_O_4_ are shown in Fig. 5(b) and the peaks are well-defined with a substantially higher current response than for NiMn_2_O_4_ alone. The increase in the current can be attributed to the improvement in the electrochemical and conductive properties of the composite, which is due to the addition of graphene.^66,67^ The results show that graphene treatment significantly enhances the charge/discharge performance of NiMn_2_O_4_, which is essential for excellent charge/discharge properties in energy storage applications.^51,68^ The correlation between the current response and the square root of the scan rate was plotted as shown in Fig. 5(c) for both materials. This plot is crucial as it substantiates the fact that the diffusion of ions at different scan rates are the primary source of the current. The NiMn_2_O_4_ and Graphene@NiMn_2_O_4_ figures are linear, indicating that ion diffusion is the main factor affecting the electrochemical properties.^69^ The charge distribution ratio found using the Dunn technique at various scan rates for both materials is shown in Fig. 5(d–e). With lower scan rates, charge distribution is predominantly that of diffusion, while with higher scan rates, it is more capacitive. For NiMn_2_O_4_, diffusion contribution is 54% at scan rate of 50 mV s^−1^ and capacitive contribution is 46% suggesting the material exhibits mainly diffusion based behaviour (Fig. 5e and f). In contrast, Graphene@NiMn_2_O_4_ shows a more favourable ratio with the capacitive contribution at 50 mV s^−1^ being 69% (compared to 31% diffusion), highlighting the improved capacitive performance of the composite material.^34^ The improved capacitance performance is mainly due to graphene, which enables increased electrochemical performance due to its ability to move electron and ion mobility.^60,70^ The change from low to high capacitive behavior indicates that the addition of graphene results in a more efficient energy storage device particularly at higher scan rate.^71^ The enhanced surface area and conductivity provided by graphene results in better electrochemical kinetics and enhanced ion and electron transport, thus enhancing this enhancement.^16^

**Fig. 6:**
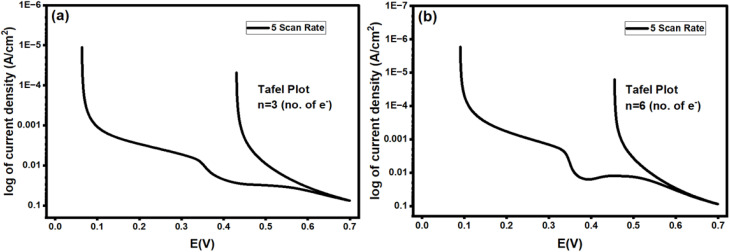
(a and b) Tafel polarization curves at 5 mV s^−1^ of NiMn_2_O_4_, and NiMn_2_O_4_@Graphene.

The Tafel plots for NiMn_2_O_4_ (6(a)) and Graphene@NiMn_2_O_4_ (6(b)) are shown, respectively, at a scan rate of 5 mV s^−1^. The cathodic slope of the Tafel polarization curve gives valuable information on the electrochemical energy storage kinetics and charge transfer behavior of the electrode material. The cathodic Tafel slope is given by the expression −2.3RT/((1 − *α*)*nF*),^72^ where *R* is the universal gas constant (J mol^−1^ K^−1^), *T* is the absolute temperature (K), *F* is the Faraday constant (C mol^−1^), *α* is the charge transfer coefficient or symmetry factor, and *n* is the number of electrons, which participate in the electrochemical reaction. In this study, the symmetry factor (*α*) was assumed to be 0.5, which is commonly used for reversible electrochemical systems.^73^ The Tafel slope of NiMn_2_O_4_ is 3 as shown in Fig. 6(a), which corresponds to the number of electrons involved in the reaction.^74^ The less steep slope suggests that the electrochemical process of NiMn_2_O_4_ has been limited by reduced electron transfer kinetics. In contrast, the Tafel slope of Graphene@NiMn_2_O_4_ shown in Fig. 6(b) is 6, which is greatly enhanced from that of NiMn_2_O_4_. The increase in Tafel slope further supports the notion that graphene improves the electrochemical activity of the material by ensuring that the charges are transferred much faster during charge/discharge cycles.^75^

**Fig. 7:**
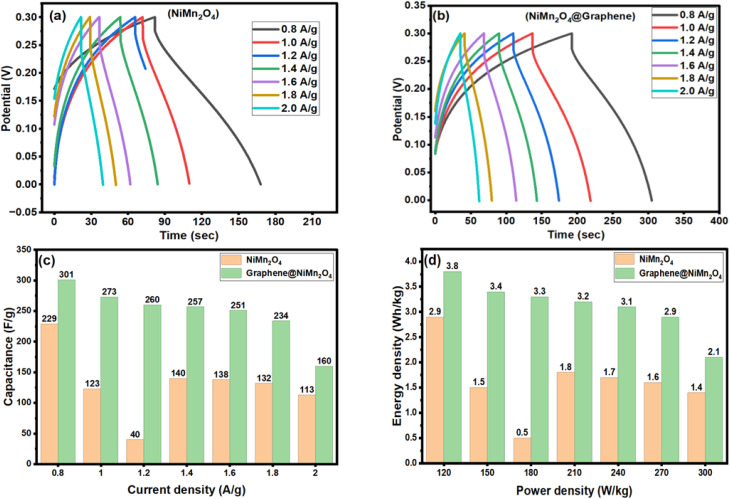
(a and b) GCD curves, (c) verses between measured capacitance *vs.* current density, (d) Ragone plot of NiMn_2_O_4_, and NiMn_2_O_4_@Graphene.

The galvanostatic charge discharge (GCD) peaks of NiMn_2_O_4_ and Graphene@NiMn_2_O_4_ in different current densities (0.8 to 2.0 A g^−1^) are shown in Fig. 7. The GCD curves are used to gain insight into the charge/discharge characteristics of the material, which include aspects related to capacity retention, energy density and power density, considered to be key parameters of the performance of energy storage devices.^75,76^ The GCD curves of NiMn_2_O_4_ are shown in Fig. 7(a) which has a typical charge/discharge behavior and reduced discharge time at high current density, indicating low stability of the material at high current rates. A more stable discharge mechanism can be observed at lower current densities (0.8 A g^−1^), with longer discharge times. In contrast, Fig. 7(b) for Graphene@NiMn_2_O_4_ presents very long discharge times, even at 2.0 A g^−1^, due to the enhanced conductivity and structural reinforcement effect that occurs when the graphene is added to the NiMn_2_O_4_ structure, thereby improving the material's durability and rate performance. The parameters, *C*_sp_ (F g^−1^), *E*_d_ (Wh kg^−1^) and *P*_d_ (W kg^−1^) are defined by attached formulas:^66^4
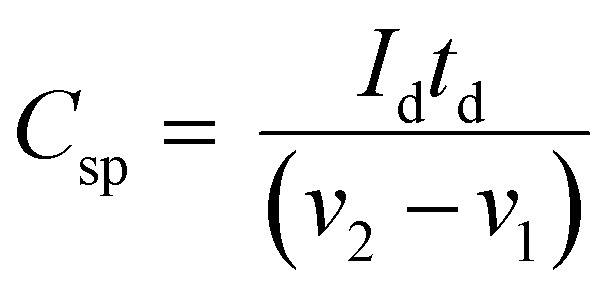
5
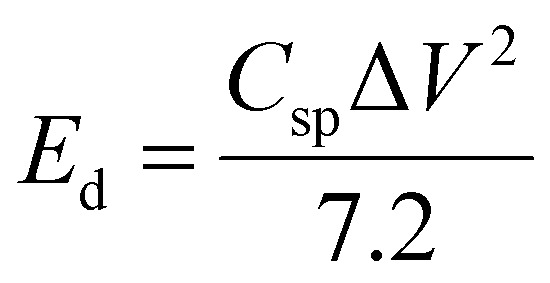
6
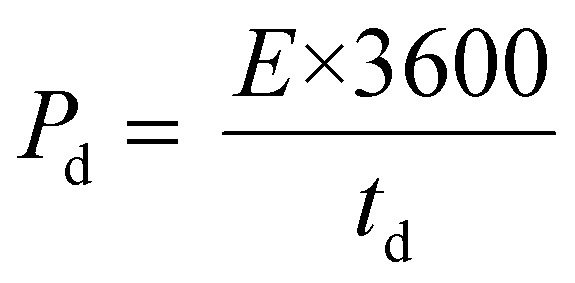


The discharge current is indicated by *I*_d_ (A g^−1^), *t*_d_ (sec) signifies the discharge time, and Δ*V* (V), is the potential window applied. Graphene@NiMn_2_O_4_ shows a maximum capacitance of 301 F g^−1^ at 0.8 A g^−1^ in contrast to 228 F g^−1^ of NiMn_2_O_4_, and has a better response at higher current densities (Table 2).^16^ Graphene@NiMn_2_O_4_ achieves a capacitance of 160 F g^−1^ under 2.0 A g^−1^, while NiMn_2_O_4_ only reaches 40 F g^−1^, showing that graphene contributes to an appreciable improvement in the rate capability (Fig. 7(c)). Indeed, at the same current density of the NiMn_2_O_4_, the maximum energy density of Graphene@NiMn_2_O_4_ is 3.8 Wh kg^−1^, which is significantly higher than that of NiMn_2_O_4_ (2.9 Wh kg^−1^), as shown in Fig. 7(d), highlighting the outstanding energy storage potential of the graphene composite.^77^ The addition of graphene to the NiMn_2_O_4_ matrix improves the conductivity, structural stability and electrochemical stability of the material. Graphene acts as a conductive substrate which allows for faster electron transport pathways and reduces total resistance.^78^ This results in improved electrochemical performance reflected in the high values of capacitance and energy density at higher current densities. As a result of graphene incorporation, the electrochemical performance of NiMn_2_O_4_ is significantly enhanced, making it an outstanding material for energy storage applications, especially high power applications.^79^

**Table 2:** Calculated parameters from the GCD curves for both samples

*I*_d_ (A g^−1^)	*C*_sp_ (F g^−1^) NiMn_2_O_4_	*C*_sp_ (F g^−1^) Graphene@NiMn_2_O_4_	*E*_d_ (Wh kg^−1^) NiMn_2_O_4_	*E*_d_ (Wh kg^−1^) Graphene@NiMn_2_O_4_
0.8	229	301	2.9	3.8
1.0	123	273	1.5	3.4
1.2	40	260	0.5	3.3
1.4	140	257	1.8	3.2
1.6	138	251	1.7	3.1
1.8	132	234	1.6	2.9
2.0	113	160	1.4	2.1

**Fig. 8:**
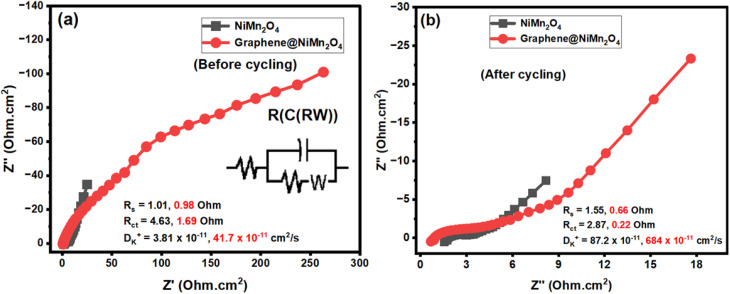
(a and b) EIS (before and after cycling) of NiMn_2_O_4_, and NiMn_2_O_4_@Graphene.

The electrochemical impedance spectroscopy (EIS) results of graphene and Graphene@NiMn_2_O_4_ before and after cycling are presented in Fig. 8. The initial Nyquist plots of NiMn_2_O_4_ and Graphene@NiMn_2_O_4_ are shown in Fig. 8(a). The high frequency part of the graph shows the charge transfer resistance (*R*_ct_) while the low frequency part refers to the Warburg impedance, which comes with the process of ion diffusion (Table 3).^80^ The charge transfer resistance (*R*_ct_) of NiMn_2_O_4_ is 4.63 Ω and the ionic diffusion coefficient (*D*_K_^+^) is 3.81 × 10^−11^ cm^2^ s^−1^. On the other hand, Graphene@NiMn_2_O_4_ shows a significantly lower *R*_ct_ value that is 1.69 Ω, and also a slightly higher ion diffusion coefficient (*D*_K_^+^ = 41.7 × 10^−11^ cm^2^ s^−1^).^81^ This suggests that the charge transfer mechanism and charge diffusion mechanism of NiMn_2_O_4_ are improved by incorporating graphene as compared to pure NiMn_2_O_4_. The impedance decreased after successive charge–discharge cycles as shown in Fig. 8(b) for the cycling process (electrochemical performance and stability), following the cycling process each sample display diminished impedance. After cycling, the charge transfer resistance (*R*_ct_) of NiMn_2_O_4_ decreases to 2.87 Ω and the ion diffusion coefficient increases to 87.2 × 10^−11^ cm^2^ s^−1^, indicating higher conductivity and ion mobility. However, this is large enhancement when it is modified by Graphene@NiMn_2_O_4_, the *R*_ct_ decreases to 0.22 Ω and the ion diffusion coefficient increases to 684 × 10^−11^ cm^2^ s^−1^. This means that the composite material gains a great advantage from the combination of NiMn_2_O_4_ and graphene, resulting in faster diffusion of ions in the material and better charge transfer after cycling.^82^

**Table 3:** Calculated parameters from the EIS for both samples

Materials		*R*_s_ (Ω)	*R*_ct_ (Ω)	*D*_K^+^_ (cm^2^ s^−1^)
NiMn_2_O_4_	Before cycling	1.01	4.63	3.81 × 10^−11^
Graphene@NiMn_2_O_4_		0.98	1.69	41.7 × 10^−11^
NiMn_2_O_4_	After cycling	1.55	2.87	87.2 × 10^−11^
Graphene@NiMn_2_O_4_		0.66	0.22	684 × 10^−11^

## 4. Conclusions

In conclusion, NiMn_2_O_4_ and Graphene@NiMn_2_O_4_ (1 : 1) composites were successfully synthesized *via* a hydrothermal method and systematically investigated for supercapacitor applications. Structural analysis confirmed the successful formation of crystalline spinel NiMn_2_O_4_ and its effective hybridization with graphene. XRD results revealed that graphene incorporation increased the crystallite size from 21.4 nm to 28.1 nm, providing more electrochemically active sites and improving charge storage capability. Raman spectroscopy verified the strong interaction between graphene and NiMn_2_O_4_, which enhanced electron transport behavior. SEM analysis demonstrated improved particle dispersion, interconnected porous morphology, and enhanced surface characteristics in the Graphene@NiMn_2_O_4_ composite, enabling effective electrolyte penetration and quick ion diffusion. Electrochemical investigations including CV, GCD, and EIS confirmed the superior electrochemical performance of Graphene@NiMn_2_O_4_, delivering a high specific capacitance of 301 F g^−1^ at 0.8 A g^−1^ and an energy density of 3.8 Wh kg^−1^, compared to 229 F g^−1^ and 2.9 Wh kg^−1^ for pristine NiMn_2_O_4_. Moreover, the composite exhibited lower charge transfer resistance and enhanced ion diffusion kinetics, demonstrating the synergistic effect between graphene and NiMn_2_O_4_. These findings suggest that Graphene@NiMn_2_O_4_ is a promising electrode material for next-generation high-performance energy storage systems. Future work will focus on optimizing the composite architecture, improving long-term cycling stability, and exploring scalable fabrication strategies for practical commercial applications.

## Conflicts of interest

The authors declare that there is no conflict of interest regarding this study.

## Data Availability

Data supporting the findings of this research are available upon reasonable request from the corresponding author.
